# Construction of a Generic and Evolutive Wheel and Lexicon of Food Textures

**DOI:** 10.3390/foods11193097

**Published:** 2022-10-05

**Authors:** Caroline Bondu, Christian Salles, Magalie Weber, Elisabeth Guichard, Michel Visalli

**Affiliations:** 1 CSGA (Centre des Sciences du Goût et de l’Alimentation), CNRS, INRAE, Institut Agro, Université de Bourgogne-Franche Comté, F-21000 Dijon, France; 2 BIA (Unité Biopolymères, Interactions, Assemblages), INRAE, F-44000 Nantes, France; 3 ChemoSens, CSGA, F-21000 Dijon, France

**Keywords:** sensory analysis, ontology, classification, hierarchy, sensory descriptors, geometrical attributes, conformation attributes, mechanical attributes, fair principles

## Abstract

In the context of data management and processing, food science needs tools to organize the results of diverse studies to make the data reusable. In sensory analysis, there are no classification or wheel of textural attributes that can be used to interpret the results of sensory studies. Research from the literature and databases was used to elaborate a list of attributes related to texture. With the help of a group of experts in food texture, work on these attributes and the related concepts was conducted to classify them into several categories, including intensity levels. The classification was represented as a texture wheel, completed by a generic lexicon of definitions of texture concepts. The work can be useful as a reference in texture attributes related to foods, and thanks to implementation in a general ontology based on food processing and observation, it can help query and interpret texture-related results from sensory studies.

## 1. Introduction

Food texture is an important parameter for understanding consumer perception and preferences [1,2,3]. Most often, texture is studied by using sensory-analysis methods [4] that focus on specific product characteristics. However, with the rise of open science and data-driven approaches, several authors have tried to draw more generic and high-level conclusions related to foods’ sensory properties. Penicaud et al. (2019) compiled data from different studies to relate the transformation process, eco-design, composition, and sensory quality in cheeses [5]. Guichard et al. (2021) did the same to study relationships between cheese composition and rheological and sensory properties [6]. More generally, with an increasing amount of data available, several studies examined the aggregation of data for the purpose of meta-analyses [7,8,9,10]. Such approaches require structuration and standardization of data and vocabulary for compliance with FAIR principles (Findability, Accessibility, Interoperability and Reusability) for scientific data management and stewardship [11]. However, although there are many classifications specific to product spaces proposed for aroma or mouthfeel attributes [12,13,14,15,16,17,18,19,20,21,22,23,24], none of them was specially designed for texture attributes. Moreover, the available lexicons mainly focus on aromas and not on texture descriptors [25]. 

Defining texture categories is a first step toward data standardization. As a first attempt to define texture categories, Szczesniak (1963) distinguished two aspects of texture: physical structure and the way in which the material is handled and felt in the mouth [26]. Szczesniak established three main categories of texture attributes: mechanical attributes, geometrical attributes and other attributes related to fattiness and moisture. Later, she refined the categories and defined texture as ‘the sensory and functional manifestation of structural, mechanical and surface properties of foods, detected by sense of vision, the ear, the touch and the kinesthesia’ [27]. These works contributed to the establishment of the ISO 5492:2008 and ISO 11036:2020 standards [28,29] that distinguish similar categories of texture. Indeed, both include 17 categories, namely hardness, cohesiveness, fracturability, chewiness, gumminess, viscosity, consistency, elasticity, adhesiveness, heaviness, denseness, granularity, conformation, moisture, dryness, fattiness and effervescence. Inside these categories, when possible, the attributes were associated with an intensity level, from ‘absence’ to ‘very high’. More recently many textures have been described in a complete guide to the textural properties of traditional and popular foods from many countries of the world [30]. However, in this guide, textures are described according to specific foods, and not all foods are considered. 

Texture is dependent on food oral processing (FOP) and, thus, is intrinsically related to FOP steps. In the sensory domain, Cairncross and Sjostrom (1950) [31] and later Brandt et al. (1963) described, with the method ‘Texture Profile Analysis’ (TPA) [32], different steps in texture perception during sensory evaluation, namely ‘first bite’, ‘chewing’ and ‘residual’. Other steps were introduced later: ‘appearance’ (visual evaluation) and ‘hand feel’ [33], ‘swallow’ and ‘after swallowing’ [34]. These FOP steps are important to consider because the texture properties of the food bolus change during the chewing process and over time. For example, the particle size decreases [35], and the chewing activity is adjusted over time [36], under the influence of saliva [37]. 

This work is part of TransformON, an ontology aiming at covering all areas of knowledge generated on the itineraries of construction/deconstruction of the quality of food and bioproducts in connection with the benefits/risks for human health and the environment. It constitutes a domain ontology on the research fields of the National Research Institute for Agriculture, Food and Environment (INRAE) Division ‘Transform’, dealing with Food, Bioproducts and Waste. An ontology [38] is a structured set of knowledge in a specific domain that always has two parts: a conceptual part, defining concepts and relationships between these concepts; and an instance part, which contains the data. In the conceptual part, introducing semantics is necessary because concepts inside are linked by semantic links expressing an identity relationship that is valid in the context of the ontology. Considering that several texture attributes can describe the same concept [35] or that one texture attribute describes several concepts, semantic links can be used in the texture classification to link two texture attributes that are considered synonymous [39]. This work on semantics can be implemented by defining a lexicon to precise definitions and to justify synonymous links [40]. 

Concepts in an ontology are organized as a thesaurus (a type of dictionary in which words with similar meanings are arranged in groups [41]), and the idea of this work was (i) to develop a large lexicon of texture attributes with their synonyms and definitions depending on FOP steps in French and in English and (ii) to build a generic classification of these texture attributes, including categories and intensity levels represented as a wheel. The results of this work will be accessible as an open access dataset which could be updated thanks to versioning.

## 2. Materials and Methods

### 2.1. Identification of Attributes

Elaborating a lexicon of texture attributes for a specific product usually includes several steps: selection of the panel; selection of the samples representative of the product space; development of the protocol, followed by the panelist to evaluate the samples; training of the panel; analysis of the results; and final selection of the most appropriate attributes to describe the product space [42]. However, these steps are not adapted to build a generic texture lexicon: the objective is not to select only the most representative attributes but to have as many attributes as possible to cover all product spaces. Therefore, instead of relying on a sensory evaluation approach, research in the literature (RL) was conducted to identify published texture lexicons. The word ‘lexicon’ was searched on the Web of Science website (available online: https://www.webofscience.com/ (accessed on 11 August 2022)) and filtered to the ‘food science technology’ subject area. From this list, texture attributes and definitions were selected. 

In addition to the RL, two internal INRAE sources were used to retrieve other texture attributes: datasets from the BaGaTel database [43] and datasets involving Free-Comment collected during the PhD of Benjamin Mahieu (PhD BM) [44] The second source was chosen because it contains non-standardized vocabulary about various product spaces collected with consumers.

### 2.2. Grouping of Attributes into Concepts, Categories, Intensity Levels and FOP Steps 

As a first phase, each attribute was translated from French to English or from English to French, using the Cambridge dictionary or the website WordReference.com (available online: https://www.wordreference.com/ (accessed on 11 August 2022)).

Then the second phase consisted in gathering, under the same concept [45], attributes that have the same meaning according to their definitions. The most cited attribute in the literature among several synonym attributes was chosen as the name of the concept. 

**Figure 1:**
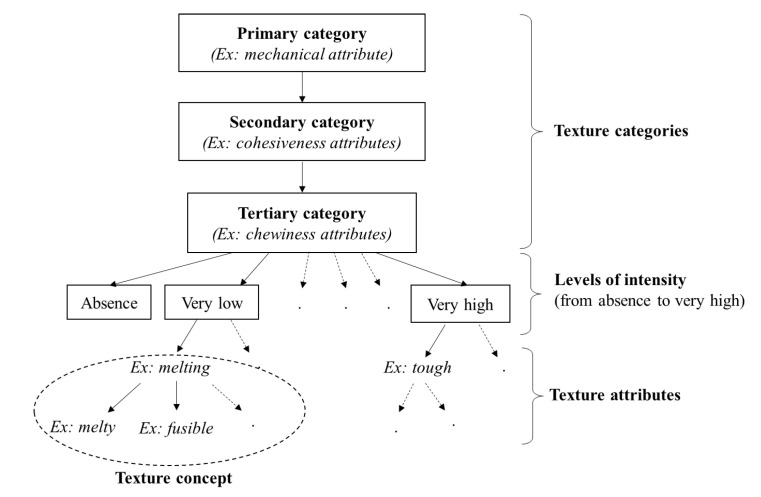
Organization of texture categories inspired by ISO 5492:2008 standard.

The third phase focused on creating general categories (primary, secondary and tertiary categories) and levels of intensity of texture concepts. The ISO 5492:2008 standard provided the basis for this classification, which presents the same categories as the ISO 11036:2020 [28,29], as represented in Figure 1. 

Figure 1 shows an example of classification for the ‘chewiness attributes’, the child of ‘cohesiveness attributes’, which is itself the child of ‘mechanical attribute’. Melting is associated with very low chewiness. It is synonymous with ‘melty’, ‘fusible’ and others. The ISO 5492:2008 categories were chosen because they could include a wide range of attributes for the purpose of genericity and exhaustivity. 

The fourth phase consisted of distinguishing different types of measurements for each concept according to the way they were evaluated during the sensory analysis (mode of evaluation) or according to the FOP steps (temporality). This step enables us to define texture concepts more precisely in the lexicon. 

**Figure 2:**
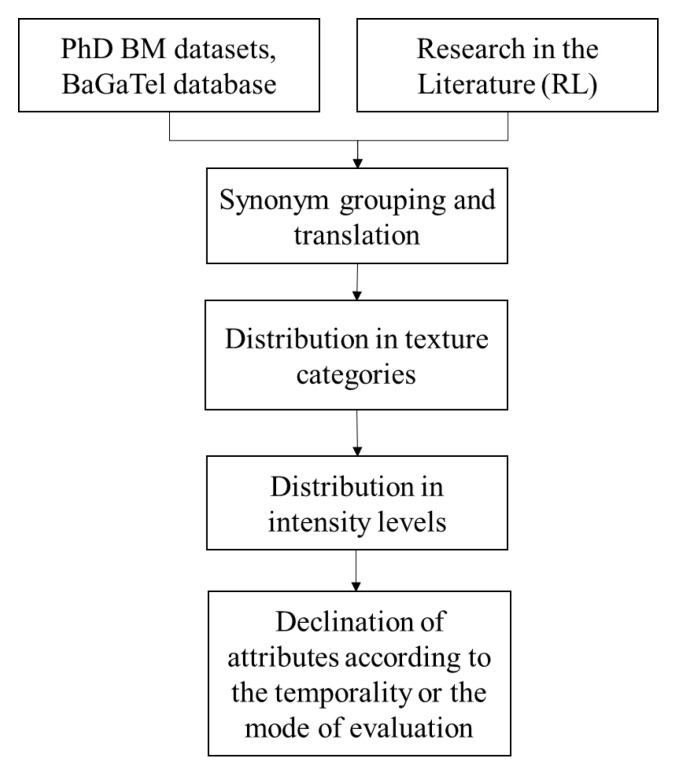
Scheme of attribute grouping steps (origin of attributes, grouping into categories, intensity levels and declination).

All of these steps are summarized in Figure 2.

### 2.3. Validation of Attribute Classification by Group of Experts 

Twelve experts from the Center for Taste and Feeding Behaviour (Centre des Sciences du Goût et de l’Alimentation (CSGA) in French) in Dijon agreed to contribute to the classification. In a lexicon-development process, the selected experts provided advice thanks to their expertise in sensory analysis on many product types [42]. However, for such a generic texture-attribute lexicon, the experts did not receive any training sessions to recognize one texture attribute from another; only their knowledge and background enabled them to classify the texture attributes. They were either researchers or teacher–researchers in FOP/food science, Food Technol. engineers, food oral-processing research engineers or research technicians in sensory analysis.

**Figure 3:**
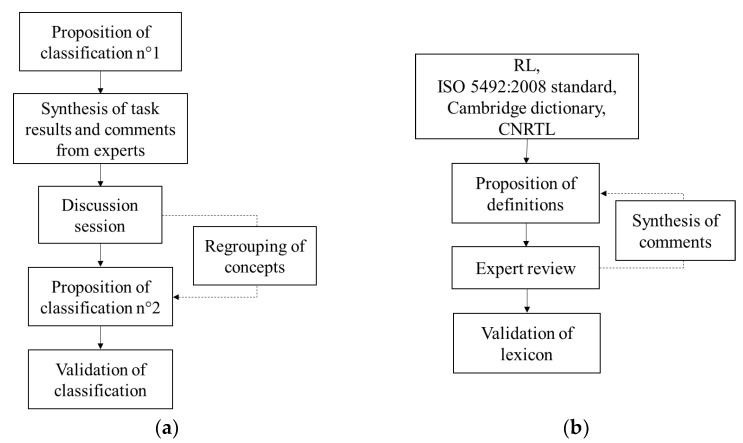
Steps of work with group of experts to validate (**a**) classification of attributes and (**b**) lexicon.

The experts received the proposal of classification as described in Section 2.2. Individually, they had to approve or modify the classification (when a suggestion exists) or classify attributes (when no suggestion exists) at different levels: category, intensity level and declinations (evaluation mode and temporality) of each attribute. They were asked to make any comment they judged appropriate. The results of this individual task were synthetized to identify the attributes that were not consensually classified by experts. If more than 10 experts out of 12 agreed, then we considered that a consensus was reached; otherwise, the attribute was submitted to group discussion. Then a two-hour group discussion session was set up to identify reasons for disagreement. Following this session, based on their feedback, the experts received a second proposal of classification to be validated. Each expert had the possibility to update the classification, and in case a new modification was proposed by one of them, all experts were informed and had to approve it. The modification was made only if more than 10 experts agreed. All steps are summarized in Figure 3a.

### 2.4. Construction of Lexicon and Validation by Group of Experts

For each concept, the lexicon provides, in English and in French, the principal attribute, the synonyms (secondary attributes) and a definition. A first set of definitions was formulated thanks to the RL, ISO standards, Cambridge dictionary or website of the National Textual and Lexical Resource Center (CNRTL) (available online: https://www.cnrtl.fr/ (accessed on 11 August 2022)). This proposition was individually reviewed by the group of experts (three reviewers per definition). The lexicon underwent several modifications, as several iterations were necessary to make the experts agree on the definitions. All steps are summarized in Figure 3b.

### 2.5. Representation of Classification by a Wheel

In the end, the classification was represented by a hierarchical wheel. The organization of the wheel is as follows: the first, second and third inner circles correspond to the primary, secondary and tertiary categories, respectively. The texture concepts are in the external circle, and the color code depends on the level of intensity. The darker the color, the higher the level in the category. White means that the concept was classified with no level of intensity inside the category. The declination of the concepts according to the evaluation mode and the temporality do not appear in the wheel, which remains for generic use. 

## 3. Results

### 3.1. Results of RL: Number of Attributes by Food Category 

A total of 340 articles and reviews were retrieved thanks to the RL. Among them, 66 included texture attributes, with most of them belonging to food lexicons (6 generic, 60 specific). The food categories covered by these lexicons were chosen according to the FoodEx2 classification [46]. The number of lexicons per category and number of texture attributes or texture categories collected in these lexicons are given in Table 1. 

### 3.2. Establishment of Texture Attribute Categories

Among the categories of texture attributes found in the 66 lexicons, several were cited many times. Table 2 includes the names of the texture-attribute categories mentioned at least three times.

A large number of categories were used in lexicons, and they were gathered into the three-level categories (three levels) described in Section 2.2. In addition to the 17 categories of the ISO standard, one category, ‘homogeneity attributes’, and three tertiary categories, ‘inside conformation’, ‘global shape’ and ‘surface conformation’, were created. They, respectively designate the homogeneity of the body and surface of a product, conformation of the material inside the product, general shape of the product and surface-texture conformation of the product. The final classification is presented in Table 3. Texture attributes were distributed in these categories afterward. 

### 3.3. Texture Attributes Used to Establish Concepts of Texture

Thanks to the RL, PhD BM datasets, and BaGaTel Database, 343 texture attributes were identified and then grouped into concepts. 

For example, the attributes ‘sticky’ and ‘adhesive’ are synonyms, but ‘sticky’ is more cited than ‘adhesive’ (see Supplementary Materials Table S1); therefore, ‘sticky’ was chosen as the preferred name, and ‘adhesive’ was designated as the alternate, both representing the same concept. The texture attributes identified in the RL standardized as adjectival forms and mentioned more than three times are presented in the Supplementary Materials. They can be interpreted as the most common texture attributes used in lexicon and sensory analyses. However, for the purpose of completeness, all 343 identified attributes were referenced in the final classification. 

### 3.4. Attribute Classification and Validation by Group of Experts 

Among the 172 concepts, 148 were preclassified and presented to experts for validation. For the remaining 24 concepts, no obvious classification was possible; thus, no suggestion was made to the experts. Concerning the tertiary category ‘conformation attributes’, the classification into levels of intensity was not possible. For this category, experts had the choice to distribute concepts into three categories, namely ‘inside conformation’, ‘global shape’ and ‘surface conformation’, that were created in the context of this work. 

In addition to validating or modifying the classification of concepts, the experts suggested changing the gathering of several concepts. For example, ‘aggregated’ and ‘agglomerate’ were gathered as synonyms by the group of experts. Indeed, as the distinction between these two terms does not exist in the field of material science, a distinction was recognized as not being necessary in the food sensory analysis. Moreover, some concepts were submitted to group discussion based on the experts’ individual feedback. The most difficult concepts to classify were those referring to multidimensional characteristics. For example, the concept ‘spongy’ can be interpreted as a moisture, denseness, chewiness or even elastic attribute. Its classification is not clear in the literature because it can be analyzed regarding different parameters. For this reason, it was decided to create a fourth primary category named ‘other attributes’ which designates these multidimensional attributes that cannot be classified anywhere else. This category also includes concepts referring to specific products, such as ‘brioche’ or ‘cardboard’, which do not have a dominant property. 

When possible, the decisions were taken based on definitions found in the literature and on the way experts use these terms in the field of food texture. In the absence of definition or consensus, we decided not to establish the level of intensity in the category, as is the case for ‘fattiness attributes’ category; this category has no intensity level. 

Concepts having several meanings were classified into several categories. For example, the concept ‘soft’ refers both to a product that is smooth, without roughness related to surface conformation, or to a product that is easy to compress and to penetrate related to hardness. Therefore, this concept was placed in two categories, ‘surface conformation attributes’ and ‘hardness attributes’, depending on its definition. 

Finally, concepts referring to effervescence caused issues among experts because of their link with the trigeminal effect. However, effervescence can still be described as a texture attribute according to the size and number of bubbles in the product. Only three levels of intensity were distinguished for this category. 

**Figure 4:**
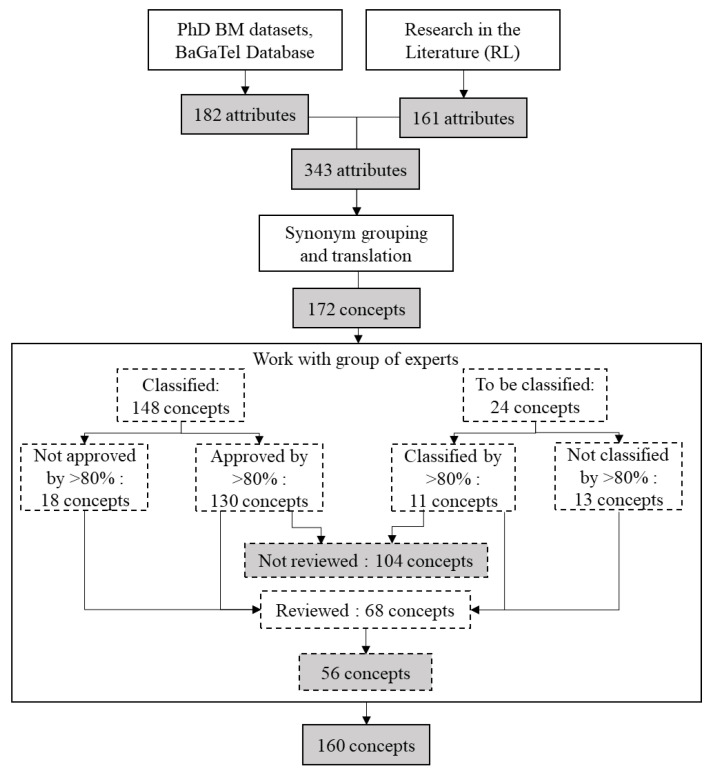
Number of texture attributes before and after step of translation and synonym grouping and after work with the group of experts.

In total, 130 concepts assigned to a category out of the 148 preclassified were validated by 80% of the experts and 11 concepts out of the 24 not preclassified were classified into a category with an agreement of 80%. However, due to the comments of the experts, 68 concepts were submitted to discussion, even though some were among the 141 (130 + 11) accepted. Ultimately, 160 concepts were classified into different levels of intensity in each category and the group of experts validated the final proposition of classification. The work with the group of experts is summarized in Table 4 and Figure 4.

### 3.5. Wheel of Texture Concepts

**Figure 5:**
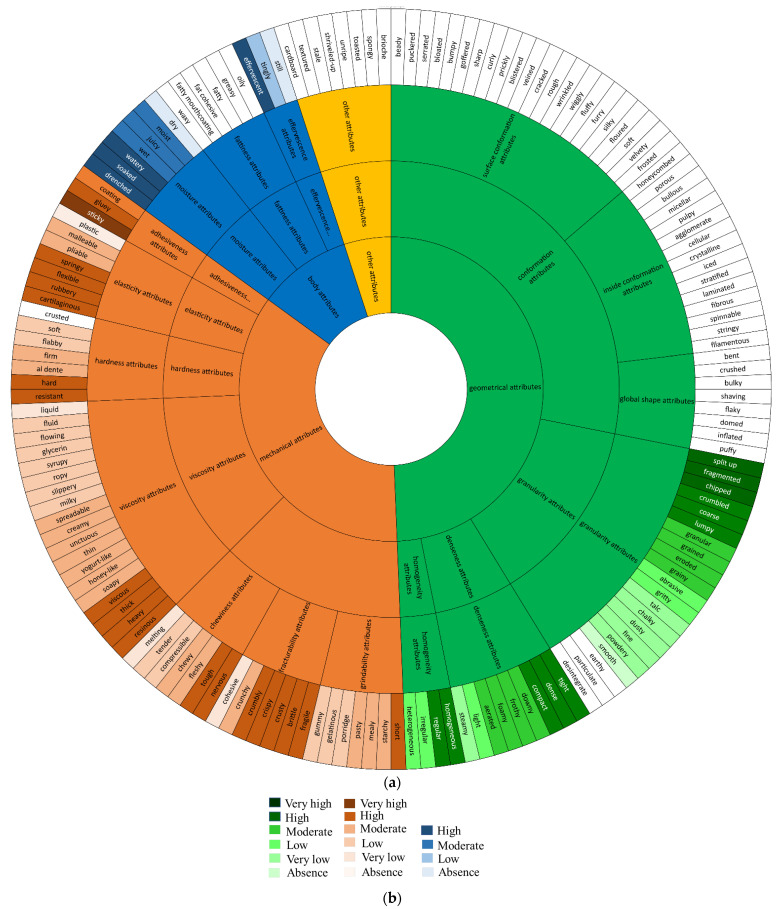
(**a**) Wheel of texture concepts and (**b**) legend.

The final classification of the concepts of texture is represented as a wheel of texture in Figure 5.

As an example, in Figure 5, the concept ‘melting’ is classified into ‘chewiness attributes’, which are the child of ‘cohesiveness attributes’, which is itself the child of ‘mechanical attributes’. The concept appears in very bright orange, signifying a very low level of chewiness. 

### 3.6. Lexicon Development 

#### 3.6.1. Definitions of Texture Attribute Categories

Considering that the ISO 5492:2008 categories were chosen for the classification, an analysis of the use of ISO definitions for these categories in the lexicons from the RL was conducted. Table 5 shows the percentages of lexicon definitions that were formulated in the same way as in the ISO standard. The number of times a definition was found for the texture category was also noted.

Looking at Table 5, we can see that six categories do not have a definition in the lexicon from the RL. For categories that were defined in lexicons, globally, there is no agreement with the ISO definition. As it was difficult to privilege the definition from one lexicon to another, the experts chose to use the ISO definition for all categories. The only one that comes from another source is the definition of denseness, which was not precise enough in the ISO 5492:2008 standard, according to the experts. A final validation of all the definitions of categories of texture attributes was performed by the group of experts.

#### 3.6.2. Definition of Texture Attributes Collected in Literature Research and Classification Depending on Evaluation Mode and Temporality during Sensory Analysis

For some attributes cited in lexicons of the RL, definitions were proposed depending on the step of tasting involved or evaluation-mode criteria. The following steps were distinguished: ‘before putting in the mouth’, ‘at first bite’, ‘during chewing’, ‘during swallowing’ and ‘after swallowing’. The following evaluation modes were considered: ‘visually’, ‘to the finger’, ‘with a utensil (spoon, knife)’, ‘to the teeth’, ‘to the lips’, ‘to the tongue’, ‘to the palate’ and ‘to the throat’. They are referenced in Table 6. They are used afterward to establish texture-concept definitions in the lexicon. When a generic definition was found without evaluation mode or temporality details, the attribute was classified as a ‘general definition’ 

#### 3.6.3. Final Lexicon

The lexicon can be found entirely in the dataset [109], which is versioned and may be implemented at any time. An overview of the definitions of the categories related to the classification of the concept ‘melting’, as well as the definitions of the concept itself and its variations, is presented in Table 7.

## 4. Discussion

The main objective of this work was to organize and define a generic food-texture vocabulary. The results of this work were produced thanks to the literature resources, and the help of experts produced a wheel and a lexicon of food-texture attributes. The discussion with experts was useful to validate the classification, agree or not agree with the level of classification from the ISO 5492:2008 standard and find a consensus to classify each attribute in one texture category and in one level of intensity inside the category when possible. The ISO 5492:2008 standard was chosen as a basis for the classification; it was inspired by the classification of Szczesniak (2002). This choice was made to pre-classify attributes, when possible, to reduce the duration of the task for the experts. This could have influenced the expert’s choices; this work is the result of a compromise a priori and required discussion among experts.

The most commonly used categories in the lexicons found in the RL (Table 2) are those established by Szczesniak [26], namely ‘cohesiveness’, ‘moisture’, ‘hardness’, ‘elasticity’, ‘adhesiveness’ and ‘denseness’. However, other commonly used categories, such as ‘firmness’, ‘smoothness’, ‘roughness’ or ‘graininess’, are not referenced in Szczesniak’s classification used for the ISO 5492:2008 or the ISO 11036:2020 standards but seem to be used extensively in food lexicons. On the other hand, there are categories established by Szczesniak that are not mentioned many times in lexicons, such as ‘fracturability’, ‘viscosity’ and ‘chewiness’. ‘Viscosity’ is the most described texture characteristic for liquid and semisolid foods and is evaluated by compressing the fluid between the tongue and the palate [110]. The hardness is more studied for solid foods [111]. Finally, some specific categories not covered by the ISO 5492:2008 standard are cited rarely in lexicons, such as ‘slickness’, ‘chalkiness’, ‘grittiness’ and 37 others. The choice of categories offered by the RL is too large to take all of them into consideration. We decided to focus on the ISO 5492:2008 standard and merge the other categories to match those from the ISO 5492:2008 standard. 

As already mentioned by Szczesniak (1963), some categories were close to each other, and this was a source of discussion between the experts. As an example, attributes from the fracturability category are close to attributes from the hardness category because the two categories are proportional in terms of individual texture evaluation using intensity scales [112]. Gumminess (or grindability) is also closely related to adhesiveness and moisture [112] and leads to difficulty in reaching an agreement between the experts. On the other hand, fracturability attributes are applicable for solid products, while viscosity attributes are applicable to semisolid or liquid products [26]. This is why the experts had no problem distinguishing attributes from these two categories.

Several terms were difficult to classify due to their multidimensional nature. Indeed, not all attributes can be described with a unique property, such as ‘unctuous’, for which it is possible to say that it is a viscosity property [113]. For example, juiciness is the combination of five perceptions: the strength with which the juice squirts out of the product, rate of juice released, total amount released during mastication, properties of the juice and effect on saliva production [114]. However, all of these perceptions are associated with the higher-level concept of moisture. The multidimensional nature is typical of the ‘integrated term’, as opposed to the ‘elementary term’, which was recognized during the discussion with experts. For example, the attribute ‘creamy’, which is related to ‘creaminess’, is an integrated term because it is the combination of the elementary terms ‘thickness’, ‘smoothness’ and ‘dairy fat’. This means that a panelist can describe a product with the attribute ‘creamy’ and another panelist can use the three attributes ‘thick’, ‘smooth’ and ‘dairy fat’ to describe the same product [42]. That is why it was necessary, in the lexicon, to define the attribute ‘creamy’ with the terms ‘thick’, ‘smooth’ and ‘dairy fat’ but still make the choice to classify it in the category ‘viscosity attributes’. 

The difficulty in synonym grouping was in deciding whether to separate or gather two similar attributes. The justification of the grouping comes from the definition of the attribute according to the literature. Moreover, the French language sometimes offers more possibilities to name a concept than the English language. Therefore, not all French attributes have a translation in English. For example, the French attribute ‘boursouflé’ has no specific translation in English. Likewise, as some texture attributes are only used in English, it is difficult to find a French translation. For example, the English word ‘spongy’ does not refer to a specific term in French; it can be ‘moelleux’, ‘spongieux’, etc. Another example is the French attribute ‘consistant’, which has no translation in English, so it was classified as a synonym of ‘compact’. As a last example, the French attribute ‘charnu’ means ‘pulpy’ for fruits or vegetables but also ‘fleshy’ for meat. As Naravane et al. stated, it is possible to classify the same concept into two different categories in the ontology [115], as it was performed for ‘soft’ in the categories ‘surface conformation attributes’ and ‘hardness attributes’. This was also the case for ‘charnu’, which was classified into two different categories: ‘inside conformation attributes’ and ‘chewiness attributes’. 

The wheel of texture produced could be compared to the wheel from Van der Stelt et al. [23] with regard to mouthfeel terminology. Applied to medical nutrition products, this wheel was developed with a group of experts but using a different methodology. Many terms from their wheel are present in our wheel, but the categories are different. In their mouthfeel wheel, ‘mouthcoating’ is a category of its own, as we classified it as an attribute. Moreover, the temporality of the sensory analysis is distinguished with the categories ‘immediately after effect’ or ‘mechanics of swallowing’, while we declined this temporality rather at a sublevel of the attribute. Finally, the wheel of texture attributes we set up is less specific than the mouthfeel wheel from Van der Stelt et al. in the way that our work was conducted starting from the texture itself rather than from the effect in the mouth. However, they added modalities for each attribute to help use the attributes in terms of the level of evaluation. The user for an attribute can choose a modality from ‘not’ to ‘very’ or from ‘slow’ to ‘fast’. In our case, the modality is at the level of the categories that propose a game of attributes from ‘absence’ to ‘very high’. Moreover, attributes may be related to a binary choice of presence or absence but also linked to a scale value chosen by the taster.

This work meets the expectation of a lexicon according to Lawless and Civille, that is, providing a classification and a standardization of sensory vocabulary that can be used by a panel of consumers or experts and companies from different countries [42]. The results of our work can be reused in different ways. It can help with sensory analysis during the test conception phase by providing an attribute list and definitions to be proposed to the panelists depending on the texture category to be studied. In this way, it can be put into perspective that certain attributes can be specialized to certain categories of products. It can also help users analyze the results of unstandardized sensory evaluations, for example, when consumers use their own vocabulary to describe product texture with Free-Comment [116]. Indeed, the classification makes it possible to read the results at the level of attributes, at the level of the subcategories (for example ‘adhesiveness’) and at the level of the primary categories (for example ‘mechanical attributes’). This classification does not include an example of a product such as in the work of Szczesniak et al. (1963), which for each level of the standard scale of a texture category, presented a product example decided by a panel [112]. Indeed, adding one product for each level of intensity inside texture categories or for each attribute could be an interesting perspective to facilitate the use of texture attributes by consumer panels. 

## 5. Conclusions

The aim of this work was to build a classification to organize the sensory texture vocabulary and define each attribute in a lexicon for generic use with most food-product spaces and categories. Indeed, weaknesses in the harmonization of texture terminology were already pointed out [117]. This work required discussion among experts to deal with the difficulties of translation, multidimensional attributes and the double meaning of attributes, which constitute the limits of this work. This work provides a reference classification and can help scientific and industrial researchers with regard to food sensory analysis at the conception and analysis phase. Nevertheless, the work of classification is never finished but can possibly be completed by adding new concepts thanks to the versioning of the dataset. 

Ultimately, the classification is implemented in a general ontology of food and properties (TransformON) to make it possible to query of heterogeneous datasets collected from different sensory methods and studies and aggregate data at different levels (attributes, intensity and categories). The levels of intensity in the classification could be efficiently described by reference samples or analogies and could be of great help for sensory analysts. This will be investigated in the future by linking our branch of ontology on the sensory descriptors to the products’ branch, which is on structuration. This work will require experimentation with consumer panels. However, this classification does not yet apply to the field of wine, which has its own vocabulary [15,104]. The final ontology will help us study links between sensory perception and other food properties such as composition, nutritional profile, technological process and consumer purchases. This work makes it possible to consider the sensory aspect in the research of healthy and sustainable food.

## Figures and Tables

**Table 1:** Number of lexicons and texture attributes or categories identified in articles and reviews from RL, presented by food category.

Product Category	Number of Lexicons	Number of Texture Attributes and Categories of Attributes	References
General	6	193	[34,47,48,49,50,51,52,53]
Vegetables and vegetable products; legumes, nuts, oilseeds, spices; starchy roots or tubers; fruits and fruit products	24	140	[9,54,55,56,57,58,59,60,61,62,63,64,65,66,67,68,69,70,71,72,73,74]
Grains and grain-based product (bread, pasta, rice)	9	109	[75,76,77,78,79,80,81,82,83]
Milk and dairy products (cheese, yogurt)	11	66	[33,84,85,86,87,88,89,90,91,92,93]
Sugar and similar, confectionery and water-based sweet deserts (chocolate, nougat, honey, ice cream)	6	60	[13,94,95,96,97,98,99]
Snacks (salty and sweet)	1	53	[94]
Meat and meat products	4	49	[100,101,102,103]
Water and water-based beverages; fruit and vegetables juices and nectars; alcoholic beverages	3	27	[104,105,106]
Composite dishes (pizza)	1	7	[107]
Seasoning, sauces, and condiments	1	1	[108]

**Table 2:** Texture-attribute categories mentioned at least three times in articles and reviews from RL.

Categories of Texture Attributes	Occurrence
Cohesiveness/cohesion	23
Moistness	22
Hardness	18
Firmness	
Elasticity/Springiness	13
Adhesiveness	
Smoothness	
Roughness	
Graininess	10
Denseness/Density	
Fracturability	7
Viscosity	
Chewiness	6
Thickness	
Stickiness	
Juiciness	5
Watery	
Friability	
Slickness	4
Chalkiness	3
Crunchiness	
Grittiness	
37 Other Categories	<3

**Table 3:** Classification of textural characteristics inspired ISO 5492:2008 standard [28].

Primary Category	Secondary Category	Tertiary Category
Body attributes	Effervescence attributes	-
	Fattiness attributes Moisture attributes	- -
Geometrical attributes	Conformation attributes	Global shape attributes *
		Inside conformation attributes *
		Surface conformation attributes *
	Denseness attributes	-
	Granularity attributes	-
	Homogeneity attributes *	-
Mechanical attributes	Adhesiveness attributes	-
	Cohesiveness attributes	Chewiness attributes
		Fracturability attributes
		Grindability attributes
	Elasticity attributes	-
	Hardness attributes	-
	Viscosity attributes	-

* Categories added to classification of ISO 5492:2008 standard.

**Table 4:** Synthesis of task of experts on texture concepts. For each category of texture attributes, we offer the number of concepts already classified and that could be modified by experts and the number of concepts supposed to be classified by experts. Results: number of concepts approved by more than 80% of experts and number of concepts classified in unique category by more than 80% of experts. Last two columns correspond to number of concepts submitted to discussion with group of experts and number of concepts validated in the end.

Categories of Texture Attributes			Individual Task Of the Experts		Task Results		Discussion	
			Number of Concepts					
			Classified	To Be Classified	Approved by More Than 80%	Classified by More Than 80%	Reviewed during Discussion	Definitely Classified
Body attributes	Effervescence attributes		5	0	3	0	3	3
	Fattiness attributes		4	1	3	0	5	6
	Moisture attributes		8	0	6	0	8	7
Geometrical attributes	Conformation attributes		38	8	37	3	12	45
	Denseness attributes		11	0	5	0	8	9
	Granularity attributes		19	2	19	0	8	21
	Homogeneity attributes		4	0	4	0	2	4
Mechanical attributes	Adhesiveness attributes		3	2	3	2	2	3
	Cohesiveness attributes	Chewiness attributes	8	1	8	1	4	7
		Fracturability attributes	7	3	7	1	3	7
		Grindability attributes	7	0	7	0	1	7
	Elasticity attributes		8	0	5	0	5	7
	Hardness attributes		10	3	9	1	3	7
	Viscosity attributes		16	4	14	3	4	19
Other attributes								8
		Total	148	24	130	11	68	160
			172					

**Table 5:** Number of times definition found for each category of texture attributes and percentage same as ISO 5492:2008 standard one.

Categories of Texture Attributes	Number of Times a Definition Was Found in the Literature Research	Percentage of the Definitions Same as ISO Definitions
Body attributes	0	N/A
Effervescence	1	0%
Fattiness/Fatty feeling	7	0%
Moisture	18	0%
Geometrical attributes	0	N/A
Conformation	0	N/A
Denseness/density	9	44%
Granularity	0	N/A
Homogeneity	0	N/A
Mechanical attributes	0	N/A
Adhesiveness	12	42%
Cohesiveness	23	7%
Chewiness	5	40%
Fracturability	7	14%
Grindability	1	100%
Elasticity	7	42%
Hardness	14	14%
Viscosity	7	20%

**Table 6:** Attributes with at least one definition found in lexicons from the literature research and related to temporality and evaluation-mode criteria. N/A indicates that the combination of evaluation mode and temporality is not applicable.

Evaluation Mode	Steps of the Texture Analysis (Temporality)				
	Before Putting in the Mouth	At First Bite	During Chewing	During Swallowing	After Swallowing
Visually	Bumpy, cracking, dry, fibrous, grainy, greasy, moist, oily, rough, crust, dense, uniform, wet	N/A	N/A	N/A	N/A
To the finger	Adhesive, firm, melt, cohesive, compact, elastic,	N/A	N/A	N/A	N/A
With a utensil (spoon, knife)	Adhesive, hard, dense, viscous	Viscous	N/A	N/A	N/A
To the lips	Fuzzy, powdery	Adhesive, slippery,		N/A	N/A
To the teeth	N/A	Cohesive, crispy, crunchy, firm, hard, soft, tender, dense, snap	Al dente, cohesive, crispy, crunchy, friable, rubbery, tender, tough, fibrous, fragile	N/A	Fibrous
To the palate	N/A		Adhesive, grainy		
To the tongue	N/A	Viscous	Elastic, oily, sandy, slicky, cohesive, crumbly, dense	Elastic,	Fatty,
To the throat	N/A	N/A	N/A	Cohesive	
In the mouth	N/A	Bubbly, dry, juicy, moist, watery, cohesive, effervescent	Chalky, chewy, creamy, doughy, fatty, greasy, gritty, gummy, juicy, mealy, melt, oily, pasty, pulpy, slimy, starchy, waxy, cohesive, crystalline, flexible	Floury, grainy,	Chalky, flaky, oily, pulpy, starchy, sticky, waxy
General definition	Brittle, bulky, elastic, fibrous, firm, flaky, fluid, grainy, mushy, particulate, pasty, plastic, slimy, soft, sticky, thick, thin, viscous, waxy				

**Table 7:** Overview of definitions of categories related to classification of concept ‘melting’ and definitions of concept and variations, presented in alphabetical order.

Parents	Texture Categories/Concepts	Synonyms	Definition
Texture attributes	Mechanical attributes	Mechanical properties	The ‘mechanical attributes’ are those related to the reaction of the product to stress. They are hardness, cohesiveness, viscosity, elasticity and adhesiveness (@source ISO 5492:2008).
mechanical attributes	Cohesiveness attributes	Cohesiveness properties	Mechanical textural attribute relating to the degree to which a substance can be deformed before it breaks, including the properties of fracturability, chewiness and gumminess (@source ISO 5492:2008).
Cohesiveness attributes	Chewiness attributes	Chewiness properties	Mechanical textural attribute related to the amount of work required to masticate a solid product into a state ready for swallowing (@source ISO 5492:2008).
Chewiness attributes	Melting	Melty, melt, meltdown, rate of melt, fusible, fusibility, deliquescence	Attribute referring to a solid product which becomes liquid almost without needing to chew (@source https://www.cnrtl.fr/ (accessed on 11 August 2022)).
Melting	Melting in the mouth		Attribute evaluated by the degree to which the sample dissolves (@source 10.1111/joss.12276) and the time required for the product to melt in the mouth when continuously pressed by the tongue against the palate (@source 10.1111/j.1745-459X.2009.00217.x).
Melting	Melting to the fingers		Attribute evaluated by the rate and degree to which the product dissolves in the hand (@source 10.1111/joss.12500).

## Data Availability

Guichard, E.; Thomas-Danguin, T.; Buchin, S.; Perret, B.; Guillemin, H.; and Salles, C. Dataset on model cheeses composition, rheological and sensory properties from six different projects exported from BaGaTel database. 2020 Portail Data INRAE, BaGaTel dataverse, V3. Available online: https://doi.org/10.15454/F40EXP (accessed on 11 August 2022). Bondu: C.; Visalli, M.; Salles, C.; Guichard, E.; Weber, M. A dataset of classification and lexicon about food textures 2022. BaGaTeL Dataverse. Recherche.Data.Gouv.Fr. Available online: https://doi.org/10.57745/DFKFYL (accessed on 11 August 2022).

## Supplementary Materials

The following supporting information can be downloaded at: https://www.mdpi.com/article/10.3390/foods11193097/s1. Table S1: Texture attributes mentioned more than three times in articles and reviews from the literature research.
