# Supplementary material for: Construction of a Generic and Evolutive Wheel and Lexicon of Food Textures
Source: Foods. 2022 Oct 5;11(19):3097. doi: 10.3390/foods11193097 (PMC9562153; doi:10.3390/foods11193097)
Supplement: Supplementary file 1 [file foods-11-03097-s001.zip › foods-1906573-Supplementary material.pdf]

Table S1. Texture attributes mentioned more than three times in articles and reviews from the literature research.

<table>
<tr><th>Adjectival form</th><th>Occurrence</th></tr>
<tr><td>Firm</td><td>12</td></tr>
<tr><td>Mealy</td><td>11</td></tr>
<tr><td>Grainy</td><td rowspan="2">10</td></tr>
<tr><td>Oily</td></tr>
<tr><td>Fat</td><td rowspan="2">8</td></tr>
<tr><td>Chalky</td></tr>
<tr><td>Gritty</td><td rowspan="4">7</td></tr>
<tr><td>Hard</td></tr>
<tr><td>Sticky</td></tr>
<tr><td>Fibrous</td></tr>
<tr><td>Moist</td><td rowspan="7">6</td></tr>
<tr><td>Particles</td></tr>
<tr><td>Smooth</td></tr>
<tr><td>Adhesive</td></tr>
<tr><td>Starchy</td></tr>
<tr><td>Fatty</td></tr>
<tr><td>Soft</td></tr>
<tr><td>Sandy</td><td rowspan="6">5</td></tr>
<tr><td>Gummy</td></tr>
<tr><td>Rough</td></tr>
<tr><td>Cohesive</td></tr>
<tr><td>Greasy</td></tr>
<tr><td>Dry</td></tr>
<tr><td>Chewy</td><td rowspan="8">4</td></tr>
<tr><td>Crumbly</td></tr>
<tr><td>Crunchy</td></tr>
<tr><td>Powdery</td></tr>
<tr><td>Spongy</td></tr>
<tr><td>Thick</td></tr>
<tr><td>Tender</td></tr>
<tr><td>Lumpy</td></tr>
<tr><td>Creamy</td><td rowspan="10">3</td></tr>
<tr><td>Dense</td></tr>
<tr><td>Flaky</td></tr>
<tr><td>Foamy</td></tr>
<tr><td>Juicy</td></tr>
<tr><td>Slimy</td></tr>
<tr><td>Snap</td></tr>
<tr><td>Thin</td></tr>
<tr><td>Pulpy</td></tr>
<tr><td>Tough</td></tr>
<tr><td>121 other attributes</td><td>< 3</td></tr>
</table>
